# Artificial intelligence in ADHD assessment: a comprehensive review of research progress from early screening to precise differential diagnosis

**DOI:** 10.3389/frai.2025.1624485

**Published:** 2025-09-02

**Authors:** Cuijie Zhao, Yan Xu, Ruixing Li, Huawei Li, Meng Zhang

**Affiliations:** ^1^Pediatrics Hospital, The First Affiliated Hospital of Henan University of Chinese Medicine, Zhengzhou, Henan, China; ^2^School of Pediatrics, Henan University of Chinese Medicine, Zhengzhou, Henan, China

**Keywords:** attention deficit hyperactivity disorder (ADHD), artificial intelligence (AI), objective assessment, differential diagnosis, biomarkers, neuroimaging, machine learning (ML)

## Abstract

Attention deficit hyperactivity disorder (ADHD) diagnosis traditionally relies on subjective assessments, which lead to challenges like symptom overlap, heterogeneity, and misdiagnosis risk. Artificial intelligence (AI), especially machine learning (ML) and deep learning (DL), offers objective assessment opportunities by processing complex multimodal data (behavioral, neurophysiological, neuroimaging, genetic). This paper reviews AI’s current applications in objective ADHD assessment, covering early screening, risk prediction, diagnostic assistance, classification, assistance in precise differential diagnosis, symptom quantification, and heterogeneous subtype identification. While AI models show significant potential in extracting objective biomarkers and improving assessment efficiency, the field faces challenges: insufficient standardized data, limited generalization, interpretability issues, potential biases, and lack of rigorous clinical validation. Future research must establish large-scale, standardized multimodal databases, develop robust, interpretable, and fair AI models, and conduct rigorous clinical translation validation to achieve responsible, precise, objective, and personalized ADHD assessment and management.

## 1 Introduction

Attention deficit hyperactivity disorder (ADHD) is characterized by persistent patterns of inattention and/or hyperactivity-impulsivity that are inconsistent with developmental level and significantly interfere with functioning or development (Faraone et al., 2021). It primarily manifests as three presentation types: predominantly inattentive presentation, predominantly hyperactive–impulsive presentation, and combined presentation (Willcutt, 2012). Globally, ADHD affects approximately 5.9% of children and adolescents, with a significant proportion persisting into adulthood, where prevalence rates range from 2.5–2.8% (Faraone et al., 2021; Salari et al., 2023). Gender distribution also shows variation, with ADHD more commonly diagnosed in boys during childhood, though this ratio tends to narrow in adolescence and adulthood, and females often present with predominantly inattentive symptoms (Mowlem et al., 2019; Faraone et al., 2021). While this review encompasses research across all age groups, particular emphasis is placed on pediatric populations (children aged 6–18 years), as this represents the critical period for early identification, intervention, and the establishment of long-term management strategies. ADHD’s clinical presentation, underlying neurobiological mechanisms, and response to interventions can vary significantly across developmental stages, with children often exhibiting more pronounced hyperactivity symptoms compared to adolescents and adults, who may present with more subtle inattentive symptoms and internalizing behaviors (Biederman et al., 2000). This developmental heterogeneity necessitates age-appropriate assessment approaches and biomarker identification strategies, which will be addressed throughout this review.

This disorder significantly impacts academic achievement, peer relationships, and social adaptation during childhood and adolescence. Its symptoms and functional impairments often persist into adulthood, causing profound and lasting adverse effects on educational attainment, career development, interpersonal relationships, daily functioning, and overall mental health (Shaw et al., 2012; Faraone et al., 2021). It may even be associated with increased risk of certain chronic physical diseases in adulthood (Cortese et al., 2016), collectively constituting a substantial personal, familial, and socioeconomic burden (Doshi et al., 2012; Shaw et al., 2012).

Current ADHD diagnosis primarily relies on DSM or ICD diagnostic criteria, which are often applied in conjunction with clinical interviews and standardized behavioral rating scales for comprehensive assessment. This process faces severe challenges due to over-reliance on subjective reports. Scale completion is susceptible to reporter bias, observational environment differences, and varying levels of understanding, with significant discrepancies often existing between different reporters, leading to insufficient objectivity in assessment results (Zysset et al., 2023). More complexly, symptom overlap and differential diagnostic difficulties are particularly prominent. ADHD’s core symptoms (especially inattention) significantly overlap with various other mental or developmental disorders, including anxiety, depression, and learning disabilities (Furman, 2005). Notably, some gifted children’s “overexcitability traits” (such as high energy, intense curiosity) are behaviorally very similar to ADHD, increasing the risk of misdiagnosis (François-Sévigny et al., 2022). Meanwhile, distinguishing Inattentive presentations from Cognitive Disengagement Syndrome (CDS) neurocognitive characteristics also highlights the ongoing complexity of differential diagnosis (Durak et al., 2025).

Furthermore, ADHD frequently co-occurs with ODD, CD, and anxiety disorders (Kaplan et al., 2001; Lange et al., 2018; Haan et al., 2022), and environmental factors [such as the COVID-19 pandemic (Gosselin et al., 2025)] may also influence disease assessment. Moreover, ADHD itself exhibits high heterogeneity, with patients showing significant individual differences in symptoms, cognitive deficits, and potential neurobiological mechanisms (Nigg et al., 2020), which existing classification criteria cannot fully capture. Finally, limitations in professional resources (such as insufficient experienced clinicians, time-consuming assessments, and uneven resource distribution) also restrict the accessibility of early identification and intervention.

Against this background, artificial intelligence (AI), particularly machine learning (ML) and deep learning (DL) technologies, has garnered significant attention due to its immense potential in processing complex, high-dimensional medical data (Handelman et al., 2018; Choi et al., 2020). AI can learn complex nonlinear patterns from multidimensional, multimodal data (such as behavioral observations, cognitive tests, neurophysiological signals EEG/eye movement, neuroimaging MRI, genomics, etc.), potentially discovering more objective and quantifiable disease-related biomarkers (Nayarisseri et al., 2021; Prelaj et al., 2024). Through integrated analysis of these data, AI shows promise in improving the accuracy, efficiency, and consistency of ADHD diagnostic assessment, and may assist in achieving earlier screening, auxiliary in more precise differential diagnosis, and more data-supported personalized assessment methods.

Given AI’s potential value and increasing research exploration in ADHD assessment, this paper aims to systematically review and analyze the current applications of AI in objective ADHD assessment. The core objective is to comprehensively demonstrate how AI utilizes multimodal data across various stages of ADHD assessment—from early screening and risk prediction to diagnostic assistance and classification, to assisting precise differential diagnosis, as well as symptom quantification and heterogeneous subtype identification. Compared to existing reviews, the unique contributions of this paper include: (1) explicitly focusing on the paradigm shift from traditional subjective methods to AI-driven “quantitative assessment support”; (2) emphasizing and deeply discussing AI’s potential and limitations in “assisting precise differential diagnosis” as a key clinical challenge; (3) conducting systematic, structured analysis and outlook on current research challenges and future directions across multiple dimensions including data, algorithms, validation and translation, ethics and society, while integrating cutting-edge concepts in the field.

**Figure 1:**
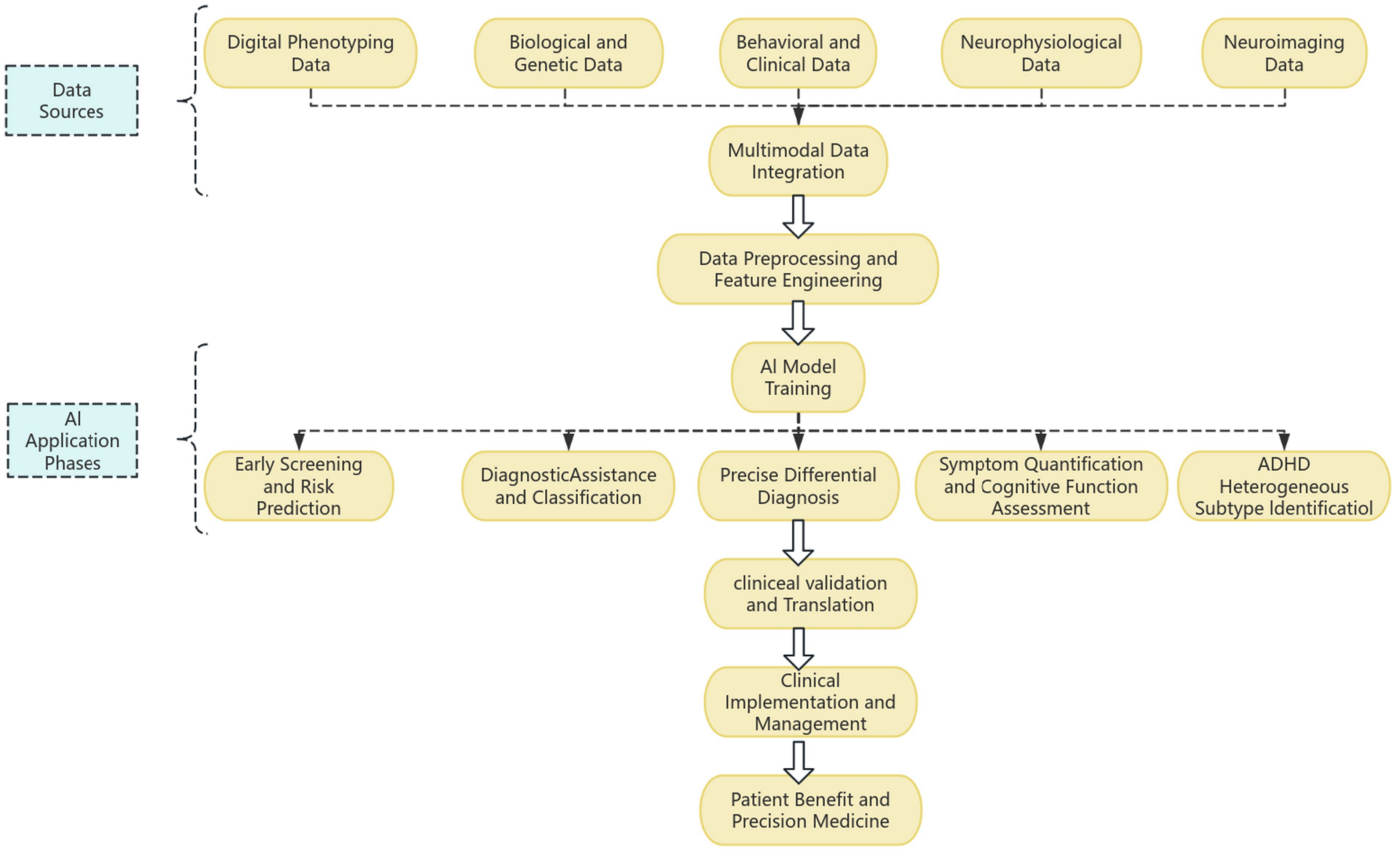
Schematic flowchart of AI-driven objective assessment for ADHD.

In the following sections, this paper will first overview the AI methodology and main data sources used for ADHD assessment, then detail AI applications in specific ADHD assessment scenarios, evaluate the performance, advantages, and limitations of existing research, followed by in-depth discussion of core challenges and future research directions, and finally conclude (Figure 1).

## 2 Data sources and modalities for artificial intelligence analysis

AI-driven objective ADHD assessment relies on diverse data inputs that reflect information from different levels, from macroscopic behavior to microscopic biology. Integrating multimodal data is considered a key strategy for improving assessment accuracy, comprehensiveness, and ultimately achieving more objective and reliable evaluation.

### 2.1 Digital phenotyping data

Digital phenotyping data offers a new, ecological, and continuous approach to ADHD assessment. Digital Phenotyping data utilizes ubiquitous personal computing devices to passively collect users’ daily behavioral data, aiming to capture more ecologically valid and objective behavioral patterns. These data include physical activity levels [step count, activity intensity (Park et al., 2023)], sleep patterns and circadian rhythms (Kim et al., 2023), social interaction patterns, screen usage habits, keyboard input patterns, and even voice features. AI can extract digital biomarkers reflecting attention, impulsivity, activity levels, mood fluctuations, and circadian rhythms from these dense, longitudinal, ecologically valid data streams (Lindhiem et al., 2022), providing possibilities for more natural, continuous, and low-burden ADHD assessment and monitoring (Loh et al., 2022; Loh et al., 2024). Studies often compare digital behavioral patterns between individuals with ADHD and neurotypical controls to identify distinctive digital phenotypes. For instance, deviations in sleep regularity, increased screen time, or characteristic movement patterns can serve as objective indicators. The experimental paradigms typically involve passive data collection through smartphone apps, wearable sensors, or digital platforms, often over extended periods to capture real-world behavior.

However, the application of digital phenotyping data also faces severe challenges, including privacy protection, data noise processing, high variability in individual behavioral patterns, and how to effectively associate digital indicators with clinical symptoms. These data are typically collected passively in individuals’ daily lives through “human-machine interfaces” such as smartphone applications and wearable sensors, and then analyzed by AI models, aiming to achieve more natural, continuous, and low-burden ADHD assessment and monitoring.

### 2.2 Biological and genetic data

Biological and genetic data provide molecular-level evidence for ADHD susceptibility and mechanistic research. Deeper objective information comes from biological and genetic data. Genomic data analysis is an important direction, where AI can be used to process high-dimensional genetic data [such as analyzing the association of single nucleotide polymorphisms (SNPs), copy number variations (CNVs), and other genetic variations with ADHD risk (Faraone and Larsson, 2019)] and build Polygenic Risk Score (PRS) models to assess individual genetic susceptibility (Ribasés et al., 2023). Studies in this area often compare genetic profiles between ADHD patients and healthy controls to identify risk variants or polygenic scores associated with the disorder.

Furthermore, some studies have begun to explore using AI to integrate other biomarkers, such as epigenetic data [DNA methylation (Silk et al., 2022)], metabolomic data [metabolite profiles in blood, urine (Predescu et al., 2024)], and even gut microbiome data (Nikolova et al., 2021) to assess ADHD likelihood. These approaches involve comparing biomarker concentrations or patterns between ADHD and control groups, often using advanced analytical techniques to identify subtle differences. Although these areas are still in preliminary exploration stages, they provide potential pathways for understanding ADHD’s complex pathophysiological mechanisms and developing new biomarkers.

### 2.3 Behavioral and clinical data

Behavioral and clinical data serve as the most foundational source for ADHD assessment, which AI can enhance for objectivity. The most traditional and fundamental data source is behavioral and clinical data, which includes standardized parent/teacher/self-rating scale scores, structured or semi-structured clinical interview records, computer-based neuropsychological test performance, and direct behavioral observation data obtained through video analysis or motion capture technology. AI models, especially when combined with other modal data, aim to extract more stable and objective assessment patterns from this information, going beyond the limitations of traditional scoring (Loh et al., 2022; Chiu et al., 2024). In research studies, these data are collected from both ADHD patients and neurotypical controls to identify characteristic behavioral patterns and cognitive deficits. Experimental paradigms often involve administering a battery of neuropsychological tests (e.g., tasks assessing attention, working memory, inhibitory control), structured clinical interviews based on diagnostic criteria (e.g., DSM-5 or ICD-11), and observations of behavior in controlled or naturalistic settings. AI is used to identify subtle deviations and complex interactions within these data, which serve as objective biomarkers distinguishing ADHD from other conditions or identifying specific symptom profiles.

### 2.4 Neurophysiological data

Neurophysiological data provides highly objective biomarkers for ADHD assessment. Neurophysiological data directly reflects brain activity or physiological states and has high inherent objectivity, making it an important source for AI to search for biomarkers. Electroencephalography (EEG) is a typical representative, where different frequency band power and their ratios in resting-state EEG, as well as functional connectivity indicators between brain regions, are widely considered potential biomarkers (Lenartowicz and Loo, 2014; Slater et al., 2022). Studies have explored brain network indices in boys with ADHD-C (Aydin et al., 2022). Additionally, the latency and amplitude of Event-Related Potentials (ERPs) are also extensively studied and used in AI classification models (Kim et al., 2021; Lutz et al., 2021). EEG data plays a crucial role in ADHD diagnosis, classification, and feature extraction, for instance, by utilizing autoencoder feature extraction and ResNet with a double augmented attention mechanism for ADHD classification (Bansal et al., 2025), or through Tunable Q-Factor Wavelet Transform for detection and classification of EEG signals (Joy et al., 2022). The impact of preprocessing and temporal segmentation on classification accuracy has also been highlighted (Garcia-Ponsoda et al., 2024). Recent research has also investigated automated EEG-based characterization of ADHD and CD using explainable deep neural network techniques (Loh et al., 2024), and the effect of methylphenidate treatment on neuro-cortical complexity in children with ADHD through embedding entropy estimations (Cetin et al., 2022).

Eye-Tracking technology indirectly reflects processes such as attentional control, impulse inhibition, and cognitive load by recording eye movement parameters during specific visual tasks (Maron et al., 2021). These parameters include saccadic latency, fixation duration, gaze patterns, and pupil dilation, often measured during sustained attention tasks (e.g., Continuous Performance Test) or visual search paradigms. Using ML to analyze these objective eye movement biomarkers has been successfully applied to assist in ADHD screening and diagnosis, by identifying distinctive patterns in ADHD patients compared to healthy controls (Yoo et al., 2024).

### 2.5 Neuroimaging data

Neuroimaging data offers objective evidence of brain structure and function for ADHD assessment. Neuroimaging data provides macroscopic objective views of brain structure and function. Structural Magnetic Resonance Imaging (sMRI) is used to measure morphological indicators such as volume, cortical thickness, and surface area of different brain regions. AI can be used for automatic segmentation of brain regions and extraction of these features, which have been found to be related to ADHD. Comparative studies between ADHD patients and control groups often reveal significant structural differences in areas like the prefrontal cortex, basal ganglia, and cerebellum, which are then used to build AI classification models (Qureshi et al., 2017; Albajara Sáenz et al., 2019).

Functional magnetic resonance imaging (fMRI), particularly resting-state fMRI (rs-fMRI), is used to study the connectivity patterns of intrinsic functional networks in the brain, such as the default mode network (DMN), salience network (SN), and central executive network (CEN). Task-based fMRI is used to observe brain activation patterns during specific cognitive tasks, like inhibitory control or working memory tasks, often comparing activation differences between ADHD and control groups. AI is commonly used to analyze complex spatiotemporal patterns and functional connectivity matrices extracted from fMRI data to distinguish ADHD patients from controls, identifying neural biomarkers indicative of the disorder (Qureshi et al., 2017; Albajara Sáenz et al., 2019; Pereira-Sanchez and Castellanos, 2021).

Diffusion Tensor Imaging (DTI) is used to assess the microstructural integrity and connectivity of white matter fiber tracts. Studies have shown that ADHD patients have altered white matter microstructure (Aoki et al., 2018; Parlatini et al., 2023), and AI can be used for fiber tract tracking and connectome analysis, utilizing DTI-derived features for classification. Biomedical images are also utilized for analysis in various medical contexts (Khekare et al., 2024).

### 2.6 Experimental paradigms and research designs

Beyond the raw data modalities, the specific experimental paradigms and overall research designs employed significantly influence the type of biomarkers identified and the generalizability of AI models in ADHD assessment. A diverse array of paradigms is utilized to probe different facets of ADHD symptomatology and underlying neurobiology.

For behavioral and neurophysiological data (e.g., EEG, eye-tracking), common paradigms include sustained attention tasks (e.g., Continuous Performance Test, CPT), inhibitory control tasks (e.g., Go/No-Go, Stop-Signal Task), working memory tasks (e.g., N-back), and reward processing tasks (Riccio et al., 2002; Owen et al., 2005; Aron, 2011). These tasks are designed to elicit specific cognitive or motor responses that are often impaired in individuals with ADHD, allowing for the extraction of objective performance metrics (e.g., reaction time variability, error rates) and physiological responses (e.g., ERP components, specific EEG frequency band power changes). The design choice (e.g., block design vs. event-related design in fMRI) influences the temporal resolution and the types of brain activity patterns that can be analyzed (Friston et al., 1998). Resting-state paradigms, where participants are asked to simply relax without engaging in specific tasks, are also widely used, particularly for fMRI and EEG, to assess intrinsic brain network connectivity and spontaneous brain activity (Broyd et al., 2009).

In neuroimaging studies (sMRI, fMRI, DTI), experimental designs typically involve comparing structural volumes, cortical thickness, or functional connectivity patterns between ADHD patients and matched healthy controls (Shaw et al., 2007; Castellanos and Aoki, 2016). Task-based fMRI paradigms in neuroimaging studies are often similar to those used for behavioral and neurophysiological assessments, but focus on mapping brain activation during the performance of these tasks. Longitudinal study designs are crucial for understanding developmental trajectories of ADHD and the long-term effects of interventions, while cross-sectional studies provide snapshots of differences at specific ages (Halperin and Schulz, 2006).

**Figure 2:**
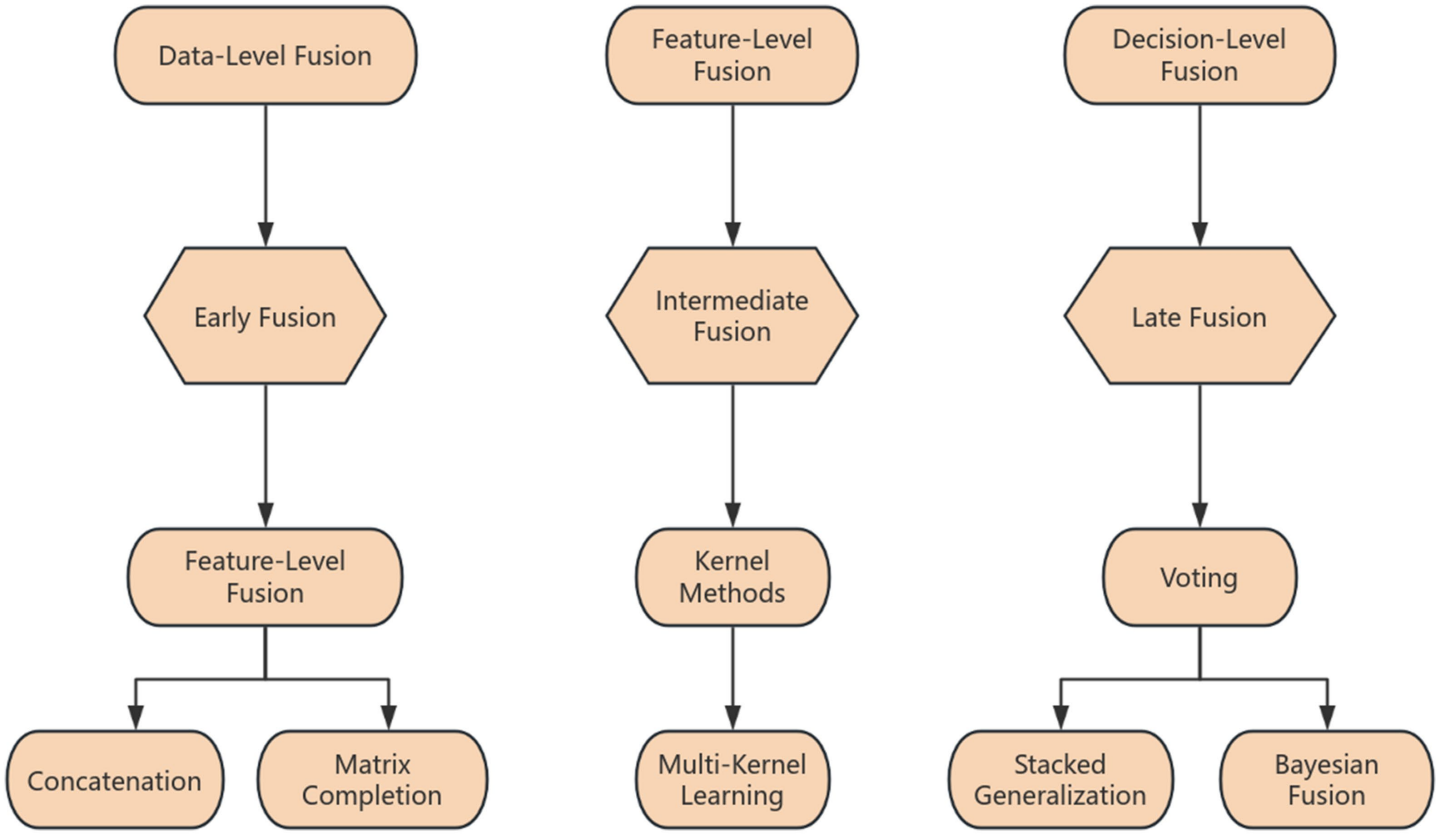
Classification framework of multimodal data fusion strategies for AI in ADHD assessment.

Data diversity and age sensitivity are critical considerations across all paradigms. Research designs must account for the heterogeneity of ADHD presentations across different developmental stages (e.g., childhood, adolescence, adulthood) and varying symptom profiles (e.g., predominantly inattentive, hyperactive–impulsive, combined) (Sonuga-Barke and Fairchild, 2012). Tailoring experimental tasks and data collection protocols to be age-appropriate ensures ecological validity and optimizes the capture of relevant biomarkers. The careful selection and rigorous implementation of experimental paradigms, along with consideration for age-specific manifestations and broader data diversity, are paramount for developing robust and clinically meaningful AI models in ADHD assessment (Figure 2).

## 3 Overview of artificial intelligence methodology for ADHD assessment

In objective ADHD assessment research, various AI are applied to analyze complex data and mine potential patterns. These technologies are primarily categorized into two branches: ML and DL. As model complexity increases, interpretability methods aimed at enhancing model transparency and credibility have gained increasing attention (Khekare et al., 2025).

### 3.1 Machine learning (ML)

Unsupervised learning and feature engineering are key to discovering potential subtypes and improving model interpretability in objective ADHD assessment. Traditional ML methods demonstrate robust performance in processing structured data and physiological or imaging features extracted through feature engineering, laying the foundation for achieving more objective and consistent assessment (Sharma and Chariar, 2024). Supervised Learning is the most commonly used paradigm, aiming to learn mapping relationships from input features to known labels. Common classification algorithms include Support Vector Machine (SVM), often employed for classifying ADHD based on neuroimaging features, and Random Forest, widely utilized for its robustness in handling diverse clinical and behavioral data, alongside XGBoost, and Logistic Regression. These are widely used to build models distinguishing ADHD patients from healthy controls (HC) or differentiating various ADHD subtypes (Jiang et al., 2017). Regression algorithms, such as Support Vector Regression (SVR) or Ridge Regression, can be used to predict continuous variables, for example, predicting specific scores on ADHD rating scales.

Unsupervised Learning aims to discover inherent structures or patterns from unlabeled data. Clustering algorithms (such as K-means, hierarchical clustering) are commonly used to explore potential subtypes defined by biological or behavioral characteristics within ADHD patient populations, helping to deepen understanding of ADHD’s high heterogeneity (Zhang et al., 2022). Additionally, when processing high-dimensional data, it is often necessary to combine Feature Selection (such as LASSO) and Dimensionality Reduction (such as Principal Component Analysis, PCA) techniques to reduce data redundancy, improve model performance, and enhance interpretability.

### 3.2 Deep learning (DL)

DL models have gained prominence for their particular expertise in automatically learning complex, hierarchical representations from raw or low-level feature data, especially suitable for processing high-dimensional, unstructured medical data. Convolutional Neural Networks (CNNs), for instance, are widely applied to analyze neuroimaging data (e.g., fMRI scans for brain activity patterns) and time-frequency representations of EEG signals (e.g., detecting abnormal brain rhythms in ADHD patients) (Quaak et al., 2021), due to their excellent performance in processing data with grid-like topological structures.

For sequence data, Recurrent Neural Networks (RNNs) and their variants, such as Long Short-Term Memory (LSTM) and Gated Recurrent Unit (GRU), can effectively capture temporal dependencies and are thus applied to analyze time series signals from EEG/MEG (e.g., identifying dynamic brain network connectivity associated with ADHD) (Chang et al., 2022; Wang et al., 2022), scan path data from eye tracking recordings, reaction time sequences in continuous cognitive tasks, and even language sequence features extracted from speech or interview texts. Transformer Models, initially successful in Natural Language Processing (NLP), have recently begun to be applied to biomedical sequence data, such as EEG signal analysis (e.g., capturing long-range dependencies in EEG rhythms for ADHD diagnosis) (He et al., 2023), fMRI time series, and clinical text analysis in Electronic Health Records (EHR) (Pillai et al., 2024). Cognitive-based ADHD detection using autoencoder-based hidden Markov models has also shown promising results (Mahesh et al., 2022).

For data representing relationships between entities, such as graph-structured brain functional or structural connectomes, Graph Neural Networks (GNNs) provide a unique analytical perspective, capable of simultaneously learning node features and graph topological structure, helping to understand ADHD-related brain network connectivity abnormalities from a network science perspective (Heck, 2021; Yang et al., 2023).

### 3.3 Machine learning (ML) and deep learning (DL) comparison

ML and DL, while both subsets of AI, possess distinct characteristics that dictate their suitability for various applications in ADHD assessment. DL, a specialized form of ML, utilizes multi-layered neural networks to learn intricate patterns directly from data. This section will compare their respective strengths, weaknesses, and common application scenarios.

Traditional ML models generally perform well with structured or tabular data. Their advantages include requiring less data for training and often offering greater interpretability, which is crucial for clinical trust and validation (Cao et al., 2023; Kerz et al., 2023). They also typically demand less computational power. However, a significant limitation is their reliance on manual feature engineering; for complex, high-dimensional, unstructured data, extensive pre-processing and feature extraction are necessary. In ADHD assessment, ML is widely applied for classification tasks using pre-extracted features from behavioral data, neurophysiological signals, or structural neuroimaging (Sharma and Chariar, 2024). They are also effective for identifying potential subtypes through clustering (Zhang et al., 2022) and for early risk prediction.

DL models excel at automatically extracting hierarchical features from raw, high-dimensional, and unstructured data, such as entire neuroimaging scans, raw EEG time series, or video data (Choi et al., 2020), significantly reducing the need for manual feature engineering. DL models scale exceptionally well with large datasets and have achieved state-of-the-art results in areas like image processing and natural language understanding. Conversely, they typically require substantial data for optimal performance, are computationally intensive, and their “black box” nature can pose challenges for clinical interpretability, though Explainable AI (XAI) techniques are actively addressing this (Amado-Caballero et al., 2023). DL is extensively used for analyzing raw neuroimaging data, processing eye-tracking scan paths, and interpreting clinical texts or speech patterns in ADHD research (Quaak et al., 2021; Chang et al., 2022; Wang et al., 2022; He et al., 2023; Pillai et al., 2024), particularly valuable when large, complex multimodal datasets are available.

Despite their differences, ML and DL are complementary. DL can serve as a powerful feature extractor, with its learned features then utilized by traditional ML models for final classification or regression. This hybrid approach can combine DL’s representation learning capabilities with ML’s interpretability or efficiency. The optimal choice between ML and DL often depends on the nature and volume of the available data, the specific task, and the importance of model interpretability within the clinical context.

### 3.4 Explainable artificial intelligence (XAI)

With increasing model complexity, especially the “black box” nature of DL models, their clinical application faces challenges in trust and adoption. Consequently, Explainable Artificial Intelligence (XAI) techniques have emerged and become increasingly important in ADHD research. Methods such as SHAP (SHapley Additive exPlanations) (Wicker et al., 2017) or Local Interpretable Model-agnostic Explanations (LIME) aim to reveal the key objective features or data patterns that models rely on for making specific predictions. By enhancing model transparency, XAI helps clinicians understand, validate, and trust model results, which is crucial for promoting reliable AI applications in mental health (Cao et al., 2023; Kerz et al., 2023). XAI not only helps clarify the basis for model decisions but may also assist in discovering new potential biomarkers, thereby deepening understanding of ADHD’s objective pathophysiological mechanisms.

## 4 Specific applications of AI in ADHD screening, diagnosis and assessment

AI have been widely applied in multiple aspects of the ADHD assessment process, demonstrating great potential from early warning to precise classification, with the core goal of improving the objectivity, accuracy, and efficiency of assessment.

### 4.1 AI-driven early screening and risk prediction

In the early stages of the process, AI is used for early screening and risk prediction. Given that early identification of ADHD is crucial for improving patient outcomes, AI models are being explored to integrate known early risk factors [such as premature birth, low birth weight, family genetic risk (Cabana-Domínguez et al., 2023), prenatal exposure factors (Ji et al., 2020)] and behavioral observation indicators during infancy [such as temperament characteristics, motor development milestones, early attention patterns (Gair et al., 2021)]. By building early risk prediction models, AI has the potential to identify high-risk children who need further professional assessment. For example, ML algorithms can analyze data extracted from parent-completed early screening questionnaires or community health records to predict the likelihood of individuals developing ADHD symptoms during school age, thereby facilitating early intervention.

### 4.2 AI-assisted diagnosis and classification

In the diagnostic phase, AI-assisted diagnosis and classification is one of its most widely applied areas. Research aims to build classifiers that can accurately distinguish ADHD patients from healthy controls (HC) based on objective data, typically using single or multimodal data. For instance, ML models like Support Vector Machines (SVMs) and Random Forests have been applied to analyze EEG signal features, achieving high accuracy in distinguishing ADHD from HC (Lenartowicz and Loo, 2014; Tenev et al., 2014; Öztoprak et al., 2017). DL models, such as Convolutional Neural Networks (CNNs) and Recurrent Neural Networks (RNNs) or Transformer Models, are increasingly used for analyzing raw neuroimaging data (fMRI, sMRI) and time-series EEG/fMRI signals, respectively, demonstrating robust performance in identifying ADHD patients and representing a current research hotspot (Riaz et al., 2020; Joy et al., 2022; Zhang-James et al., 2023; Firouzi et al., 2024; Loh et al., 2024; Taspinar and Ozkurt, 2024; Bansal et al., 2025). It is generally believed that models integrating multiple modalities can achieve better classification performance than single modalities and potentially provide more comprehensive and reliable assessment results (Qureshi et al., 2017; Riaz et al., 2018).

### 4.3 AI applications in precise differential diagnosis

AI’s application in precise differential diagnosis is particularly prominent, addressing a core challenge in current ADHD clinical assessment. Due to overlapping symptom presentations, accurately distinguishing ADHD from other neurodevelopmental/mental disorders with similar presentations or comorbidities is extremely challenging. AI technology, with its ability to analyze complex patterns, aids in achieving more precise differential diagnosis.

In distinguishing ADHD from gifted children, their behavioral presentations may be very similar, leading to misdiagnosis. AI research is attempting to assist this differentiation process through more refined data analysis. For example, using ML models to analyze parent and teacher behavioral rating scale data to identify subtle but distinctive differences in specific behavioral patterns between the two groups (François-Sévigny et al., 2022). In the future, combining more objective biobehavioral indicators and using AI for comprehensive analysis may provide more objective and reliable evidence.

For distinguishing ADHD from anxiety/depression disorders, inattention is a common symptom, increasing the difficulty of differentiation. AI technology can assist in differentiation by integrating and analyzing multiple data sources. Using AI models to analyze neuroimaging data can help find differential biomarkers (Hettwer et al., 2022). In addition, applying NLP techniques to analyze clinical interview records or social media text can help identify language patterns that reflect different potential emotional states (Thorstad and Wolff, 2019); utilizing AI models to analyze neuroimaging data (such as structural or functional connectivity patterns of the amygdala and prefrontal cortex) can help find differential biomarkers; or analyzing physiological signals such as Heart Rate Variability (HRV) can explore their potential in objective differentiation (Sun et al., 2023).

In distinguishing ADHD from learning disabilities (SLD)/developmental coordination disorder (DCD), careful differentiation is needed due to frequent co-occurrence or similar symptoms. AI models have the potential to train classifiers by integrating multiple aspects of information (such as standardized neuropsychological test results, academic performance records (Czerniak et al., 2023), objective motor coordination assessment based on video analysis (Ouyang et al., 2024), etc.) to identify characteristic presentations under different disorder patterns and improve differentiation accuracy.

Regarding the challenge of distinguishing ADHD from Autism Spectrum Disorder (ASD), although their core symptoms differ, they have high comorbidity rates and behavioral overlap. AI provides multiple approaches, including using ML/DL to analyze neuroimaging data [comparing whole-brain functional connectivity patterns or specific brain region structural differences (Eslami et al., 2020; Song et al., 2020)], or analyzing other behavioral/physiological data (such as quantifying behavioral features in social interaction videos through computer vision, analyzing social scene gaze preferences using eye tracking, etc.), aiming to discover objective and effective biobehavioral markers that distinguish the two (Moreau et al., 2023).

Furthermore, recent research has begun to focus on the challenge of distinguishing ADHD inattentive type (ADHD-RI) from cognitive disengagement syndrome (CDS) (Durak et al., 2025). Using AI to analyze detailed neurocognitive test data or specific objective neurophysiological indicators, may help identify characteristic patterns that distinguish between these two conditions and improve differential diagnostic accuracy.

### 4.4 AI quantification of symptom severity and cognitive function

Beyond classification and differential diagnosis, AI is also applied to quantify symptom severity and cognitive function, which helps more precisely track disease progression and treatment response. For example, by analyzing objective behavioral or physiological data [such as CPT performance metrics, activity levels and sleep patterns collected by wearable devices (Lindhiem et al., 2022; Kim et al., 2023)], AI models have the potential to objectively quantify the severity of individual inattention, hyperactivity, and impulsivity. This assessment may be more sensitive in capturing subtle changes than traditional subjective rating scales. Some studies also use ML models, based on objective biomarkers or other objective measurement data, to directly predict individual scores on standard ADHD rating scales (such as ADHD-RS) (Cervantes-Henríquez et al., 2022), aiming to provide more objective reference for clinical assessment.

In terms of cognitive function, AI algorithms can integrate and analyze objective performance data from multiple neuropsychological tests (especially those evaluating executive functions) (Duff and Sulla, 2015; Takahashi et al., 2024), potentially going beyond traditional single total score evaluation to more precisely characterize individual specific impairment levels and unique patterns in different cognitive subdomains, providing objective information for understanding individual differences and guiding personalized cognitive interventions.

### 4.5 AI-driven ADHD subtype identification

Finally, to address ADHD’s high heterogeneity (Nigg et al., 2020), researchers use unsupervised learning (such as clustering algorithms) and other AI methods to identify ADHD subtypes. By analyzing objective biological or clinical features (such as based on resting-state fMRI functional connectivity patterns (Miranda et al., 2021) or EEG microstate features (Alves et al., 2024)), researchers attempt to identify subgroups within the ADHD patient population that have different brain network abnormalities, cognitive deficit patterns, or treatment responses (Karalunas and Nigg, 2020). These data-driven discovered subtypes aim to go beyond traditional clinical classification and lay the foundation for achieving more precise stratified diagnosis (stratified diagnosis) and personalized interventions.

## 5 Performance evaluation, advantages and current limitations

### 5.1 Overview of existing AI model performance

Based on a comprehensive review of existing literature, AI models demonstrate encouraging performance in specific ADHD assessment tasks. For instance, in binary classification tasks distinguishing ADHD from healthy controls (HC), models utilizing high-quality multimodal neuroimaging data and advanced DL algorithms often achieve accuracy or Area Under the Curve (AUC) values of 0.8 or higher (Quaak et al., 2021; Zhang-James et al., 2023; Sharma and Chariar, 2024; Taspinar and Ozkurt, 2024). However, when facing more challenging differential diagnostic tasks [such as distinguishing ADHD from ASD (Duda et al., 2017)] or ADHD internal subtype identification (Qureshi et al., 2016; Gao et al., 2023), model performance typically decreases, although still showing potential to surpass traditional methods.

### 5.2 Potential advantages of AI in ADHD assessment

AI offers several significant potential advantages in ADHD assessment. Despite this, AI application in ADHD assessment still offers significant potential advantages. First, AI analysis is primarily based on quantifiable objective data, thereby enhancing assessment objectivity, reducing reliance on subjective reports and clinical observations, and potentially improving the stability and reproducibility of assessment results. Second, AI can rapidly process and analyze large amounts of complex data, automating parts of the assessment process (such as signal analysis, image processing), potentially improving assessment efficiency, reducing clinician workload, and shortening assessment cycles. Third, AI’s powerful pattern recognition capabilities, particularly DL, help mine potential objective biomarkers or feature combinations from high-dimensional, multimodal data that are difficult to identify using traditional statistical methods. This not only may improve diagnostic accuracy but also helps deepen understanding of ADHD’s underlying pathophysiological mechanisms. Additionally, by identifying different biological or clinical subtypes and quantifying individual specific impairment patterns across multiple dimensions, AI provides possibilities for achieving more precise personalized assessment and ultimately stratified intervention strategies (precision medicine). Finally, AI screening or monitoring tools based on mobile computing platforms such as smartphones and wearable devices have advantages of low cost, convenient deployment, and the ability to collect data in natural environments (ecological validity), potentially improving assessment service accessibility, extending to resource-limited areas, or more conveniently integrating into patients’ daily lives.

### 5.3 Limitations and challenges in current research

However, the path to applying AI in objective ADHD assessment is not smooth, and it is still in the development stage, facing numerous severe limitations and challenges that hinder the full realization of its potential and transformation into reliable clinical tools. One of the most prominent issues is data limitations and lack of standardization. Most published studies rely on small-scale, single-center datasets, with significant heterogeneity in data collection standards (such as equipment parameters, task paradigms) and preprocessing procedures across different studies, severely limiting model generalization ability and the reproducibility and comparability of research results (Zhang-James et al., 2023; Tian et al., 2024). The lack of large-scale, standardized, publicly available, and representative multicenter multimodal databases is a key bottleneck constraining the development of this field. Notably, most current AI model training datasets primarily focus on diagnosed ADHD patients and typically developing control groups, but lack systematic data collection on healthy variations (neurodiversity) that only exhibit inattentive/hyperactive traits without meeting ADHD diagnostic criteria (i.e., without significant functional impairment). This data deficiency constitutes an important confounding factor, which may lead to AI models struggling to distinguish between normal traits and the disorder, thus posing a risk of pathologizing neurodiversity. Future research urgently needs to construct representative datasets containing such ‘intermediate groups’ to improve the specificity and differentiation capabilities of AI models.

Closely related to this is the generally insufficient generalization ability and reliability of existing models. Models that perform excellently on specific internal datasets often show significant performance degradation when applied to new, independent external datasets from different populations, devices, or collection environments. This overfitting problem and domain shift effect severely limit the direct application potential and reliability of models in real-world clinical settings (Dwyer and Koutsouleris, 2022).

Another major obstacle is the poor interpretability of many high-performance AI models (particularly DL models), which are like “black boxes” with opaque internal decision logic (Cao et al., 2023). This not only hinders clinicians’ understanding and trust in model output results but also makes it difficult to gain new insights into disease mechanisms. This is one of the main barriers to clinical acceptance and adoption of models, although research on explainable AI (XAI) methods is emerging to address this challenge (Amado-Caballero et al., 2023).

Meanwhile, potential bias risks and fairness issues cannot be ignored. If training data fails to adequately represent the diversity of the target population (such as biases in age, gender, race, socioeconomic status, etc.), models may learn and even amplify these biases, leading to systematic unfairness or inaccuracy in assessment results for certain subgroups (Lee et al., 2021; Elendu et al., 2023), potentially exacerbating health inequalities.

Most critically, there is currently a severe lack of clinical validation. The vast majority of related research remains at the stage of technical exploration, retrospective analysis, or small-scale proof-of-concept, with an extreme lack of well-designed, rigorously executed prospective, multicenter studies with representative samples (such as diagnostic accuracy trials, randomized controlled trials) to evaluate the actual performance, safety, clinical utility, and cost-effectiveness of AI tools in real clinical scenarios (Rashid and Calhoun, 2020; Dwyer and Koutsouleris, 2022). The lack of high-quality clinical evidence is a core obstacle hindering the transformation of AI technology into clinical practice.

Finally, seamless implementation and integration of AI tools into existing clinical workflows also face multiple barriers in terms of technology (such as data interfaces, system compatibility), resources (such as equipment costs, personnel training), and processes (such as assessment time, report interpretation), and some methods relying on expensive equipment or complex processes have limited accessibility and scalability in resource-limited environments, which may lead to application inequality (Rashid and Calhoun, 2020; Dwyer and Koutsouleris, 2022).

A significant challenge lies in how AI evaluates and quantifies the crucial subjective criterion of “significant impairment and suffering” in ADHD diagnosis. Although AI cannot directly understand or feel an individual’s internal experiences, it can indirectly infer or quantify the degree of functional impairment by analyzing digital phenotyping data (e.g., daily activity levels, sleep patterns, screen usage habits) (Lindhiem et al., 2022; Kim et al., 2023) and behavioral data (e.g., academic performance records, social interaction frequency, objective indicators of functional impairment in multi-source behavioral reports) (Chiu et al., 2024; Ouyang et al., 2024; Czerniak et al., 2023). However, these data serve only as auxiliary evidence for clinical assessment, and their interpretation must be comprehensively judged by experienced clinicians, integrating non-structured information such as the individual’s socio-cultural background, family environment, and narrative reports. AI’s value lies in identifying objective patterns that may indicate or accompany significant impairment, rather than replacing the understanding of subjective suffering or the final clinical judgment (Lee et al., 2021; Elendu et al., 2023; Cao et al., 2023).

## 6 Core challenges and future research directions

Future research urgently needs to establish large-scale, standardized multimodal databases through international collaboration, develop robust, interpretable, and fair AI models, and conduct rigorous, transparent clinical translation validation, with the ultimate goal of achieving responsible AI application in precise, objective, and personalized ADHD assessment and management.

### 6.1 Data level: building a solid foundation and promoting secure sharing

In order to facilitate the development of robust AI models, the creation of large-scale, standardized, multimodal databases and ensuring safe, ethical data sharing are necessary measures. First, through international collaboration and multidisciplinary cooperation, we need to jointly build high-quality, large-scale, standardized multimodal databases, which are the cornerstone of the entire field’s development. The ideal database should include thousands or even tens of thousands of participants, have longitudinal tracking, multicenter sources, and good population representation, and integrate multidimensional information including behavior, cognition, neurophysiology (EEG, eye movements, etc.), neuroimaging (sMRI, fMRI, DTI, etc.), genetics, environmental exposure, and digital phenotyping. The key is to reach consensus and strictly implement data collection protocols, quality control standards, and annotation specifications to ensure data quality, promote research reproducibility and model comparability, and provide the necessary foundation for developing more powerful and generalizable AI models (Tian et al., 2024). Leveraging emerging technologies such as high-precision motion capture to quantify behavioral phenotypes is also worth exploring (Wu et al., 2025). Meanwhile, under strict compliance with ethical and privacy regulations, it is necessary to actively explore secure and compliant data sharing mechanisms and privacy protection technologies. Privacy-preserving computing technologies such as Federated Learning (Loftus et al., 2022; Brauneck et al., 2023) allow collaborative model training without directly transmitting original sensitive data, potentially breaking down data silos and improving model reliability and generalization ability by utilizing broader and more diverse data. Establishing clear data governance frameworks and access control protocols is also indispensable. Hybrid frameworks integrating blockchain, IoT, and cloud computing also offer secure and scalable solutions for healthcare data management (Kumaran et al., 2024).

### 6.2 Algorithm level: improving performance, reliability, and trustworthiness

At the algorithmic level, the core challenge lies in continuously improving AI model performance, reliability, and trustworthiness through advanced algorithms, multimodal data fusion, and considerations for interpretability and fairness. This means continuously developing AI algorithms that can better handle data heterogeneity, have strong anti-interference capabilities, and perform stably and reliably on unseen data and different populations, to enhance model reliability and generalization ability. Transfer learning, domain adaptation/generalization, adversarial training, and ML combined with causal inference are important research directions. Meanwhile, Algorithmic Fairness (Lee et al., 2021) must be incorporated as a core consideration in model design, training, and evaluation, adopting bias detection and mitigation techniques to ensure fair performance across different subgroups (classified by gender, race, etc.) and avoid exacerbating health inequalities.

Second, we should deepen multimodal fusion and information integration strategies. Given the complexity of ADHD, single-modal information is limited, requiring the development of more advanced and effective multimodal data fusion strategies to fully utilize the complementarity and synergistic effects of information from different sources, building more comprehensive and precise assessment models.

Third, we need to focus on breakthroughs in interpretability (XAI) and causal inference. Vigorously developing and applying XAI technology is a core link in winning clinical trust, promoting adoption, and potentially discovering new knowledge. Future XAI should provide explanations that conform to clinical logic, are intuitive and easy to understand, and have practical guidance for decision-making. Exploring the combination of ML with causal inference, attempting to move from association to causal exploration, may help deepen understanding of pathological mechanisms.

Finally, to adapt to mobile health applications or resource-limited scenarios, we need to develop lightweight models and explore edge computing applications, reducing model complexity through techniques such as model compression and knowledge distillation, and exploring computation on terminal devices to improve real-time performance, accessibility, and privacy.

### 6.3 Validation and translation level: rigorous evaluation and promoting clinical implementation

For AI tools to be clinically viable, rigorous and transparent clinical validation studies are essential, alongside the establishment of clear regulatory frameworks to ensure their safe and effective translation into practice. This necessitates going beyond simple internal validation and conducting rigorous, transparent, independent clinical validation studies. Large-scale, prospective, multicenter, independent external validation studies and clinical trials following recognized reporting standards [such as TRIPOD (Alewine et al., 2015), STARD-AI (Muehlematter et al., 2023)] should be designed and implemented. Meanwhile, special attention needs to be paid to bridging the gap between laboratory performance and real-world practice performance (performance gap) (Jurva et al., 2025), and conducting calibration evaluation of prediction model probability outputs to ensure their confidence accurately reflects actual risk (Benster et al., 2025).

It is also necessary to develop user-centered clinical decision support systems (CDSS), integrating validated models into user-friendly platforms or EHR systems that conform to clinical workflows, providing clear, interpretable, actionable auxiliary information, and emphasizing human-machine collaboration, with final decision-making authority resting with doctors.

Additionally, establishing clear regulatory science frameworks and approval pathways is crucial. With the development of AI/ML-driven medical devices (including SaMD), regulatory agencies (such as FDA, EMA) are improving corresponding frameworks (Pennello et al., 2021; Muehlematter et al., 2023), clarifying requirements for performance, safety, effectiveness, data quality, algorithm transparency, risk management, and post-market surveillance, providing clear paths for innovative product market access while ensuring patient safety.

### 6.4 Ethical and social level: advocating responsible innovation and ensuring universal accessibility

Addressing ethical and social considerations, such as responsible innovation, universal accessibility, and mitigating potential biases and privacy risks, is paramount for the equitable application of AI in healthcare. The primary principle is to follow strict ethical standards and data governance principles, ensuring full dynamic informed consent throughout the process involving ADHD patient data, respecting privacy rights and data autonomy, and establishing sound data security and governance frameworks to prevent risks.

Proactive identification, assessment, and management of algorithm biases and promotion of fairness (Lee et al., 2021) are necessary. At all stages of the model lifecycle, fairness metrics and bias mitigation techniques must be adopted to ensure AI tools have equal effectiveness and safety across different subgroups, avoiding technology exacerbating inequalities.

Clarification of human-machine collaboration models and responsibility attribution frameworks is needed, clearly defining AI’s role as an auxiliary tool, emphasizing that final decisions are made comprehensively by clinicians, and establishing clear accountability mechanisms.

Finally, focus on technology accessibility and bridging the digital divide is necessary. When promoting AI tools, measures should be taken to ensure the universality of technology, avoiding exacerbating health service inequalities due to equipment, technical barriers, or digital skill differences (Elendu et al., 2023).

## 7 Conclusion

AI provides unprecedented opportunities to address the many challenges in current ADHD diagnosis and assessment, particularly in improving assessment objectivity, efficiency, and achieving more precise differential diagnosis and personalized assessment. This paper systematically reviewed the current applications and research progress of AI in objective ADHD assessment, emphasizing how AI leverages multimodal data, including behavioral data, neurophysiological signals, neuroimaging features, genetic information, and emerging digital phenotyping, across various assessment stages. The significant potential of AI in tasks such as early screening and risk prediction, diagnostic assistance and classification, precise differential diagnosis, symptom severity quantification, and the identification of heterogeneous ADHD subtypes was highlighted. By integrating these diverse data sources, AI models offer a pathway to overcome the limitations of traditional subjective assessment methods, paving the way for more objective and consistent diagnostic outcomes.

However, transforming these promising AI research results into reliable clinical practice tools that can truly benefit the vast number of ADHD patients and their families in a safe, effective, and fair manner still faces severe challenges. These challenges span multiple dimensions, including obtaining, standardizing, and securely sharing high-quality data; improving algorithm reliability, generalization ability, and interpretability; conducting rigorous and sufficient clinical validation; developing effective translation strategies; and addressing comprehensive ethical, legal, and social impact considerations. These intertwined complexities necessitate a collaborative effort from the entire research, development, application, and regulatory ecosystem to effectively overcome.

Looking ahead, the future direction for AI in objective ADHD assessment is clear and promising. Substantial and synergistic progress must be made through close interdisciplinary collaboration. This includes continued efforts in building a solid, standardized data foundation, promoting responsible algorithm innovation that prioritizes interpretability and fairness, implementing rigorous and transparent clinical validation studies, and improving sound ethical and governance frameworks. Only through such concerted efforts can AI truly become a powerful, reliable, and trustworthy partner for clinicians in assessing and managing the complexity of ADHD, ultimately promoting a deeper understanding of the disorder and significantly improving the long-term well-being and quality of life for affected individuals and their families.
